# An Integrative Systems Biology Approach Identifies Molecular Signatures Associated with Gallbladder Cancer Pathogenesis

**DOI:** 10.3390/jcm10163520

**Published:** 2021-08-10

**Authors:** Nabanita Roy, Mrinmoy Kshattry, Susmita Mandal, Mohit Kumar Jolly, Dhruba Kumar Bhattacharyya, Pankaj Barah

**Affiliations:** 1 Department of Molecular Biology and Biotechnology, Tezpur University, Sonitpur 784028, India; nitaroy@tezu.ernet.in (N.R.); mrinmoy3012@gmail.com (M.K.); 2 Centre for BioSystems Science and Engineering, Indian Institute of Science, Bangalore 560012, India; susmita.mandal1894@gmail.com (S.M.); mkjolly@iisc.ac.in (M.K.J.); 3 Department of Computer Science and Engineering, Tezpur University, Sonitpur 784028, India; dkb@tezu.ernet.in

**Keywords:** gallbladder cancer, transcriptomics, differentially expressed genes, co-expression network, transcription factors, epithelial-mesenchymal-transition, cell cycle machinery

## Abstract

Gallbladder cancer (GBC) has a lower incidence rate among the population relative to other cancer types but is a major contributor to the total number of biliary tract system cancer cases. GBC is distinguished from other malignancies by its high mortality, marked geographical variation and poor prognosis. To date no systemic targeted therapy is available for GBC. The main objective of this study is to determine the molecular signatures correlated with GBC development using integrative systems level approaches. We performed analysis of publicly available transcriptomic data to identify differentially regulated genes and pathways. Differential co-expression network analysis and transcriptional regulatory network analysis was performed to identify hub genes and hub transcription factors (TFs) associated with GBC pathogenesis and progression. Subsequently, we assessed the epithelial-mesenchymal transition (EMT) status of the hub genes using a combination of three scoring methods. The identified hub genes including, CDC6, MAPK15, CCNB2, BIRC7, L3MBTL1 were found to be regulators of cell cycle components which suggested their potential role in GBC pathogenesis and progression.

## 1. Introduction

The gallbladder is a small sac-like structure located beneath the liver that forms an integral component of the biliary tract system. Gallbladder cancer (GBC) is the sixth most frequent cancer of the gastrointestinal tract worldwide. GBC is an aggressive malignancy, with rapid progression, poor prognosis and a high mortality rate resulting in an overall 5-year survival rate of only 5% [1,2]. The incidence rate of GBC is highly marked by distinct geographic and ethnic disparities. Such regional and ethnic discrepancy in the incidence rate of GBC cases indicates the differences in GBC etiology in different populations [2,3]. According to recent GLOBOCAN report (http://globocan.iarc.fr, accessed on 1 January 2018), GBC ranks in the 20th position among the most frequent cancer types, with approximately 0.2 million cases diagnosed annually. The incidence of GBC cases is highest in the Eastern Europe, East Asian country and Latin American regions, with the incidence ratio of GBC cases being the highest in South American countries such as Chile, Bolivia and Ecuador and Asian countries, mainly including Korea, India, Japan and Pakistan [4,5].

GBC is an orphan disease and its etiology is multifactorial. The pathological spectrum of GBC mainly progresses from metaplasia to dysplasia with subsequent carcinoma-in-situ and cancer metastasis suggesting that an epithelial mesenchymal transition (EMT) event might be an important phenomenon in GBC development. The detailed molecular mechanism of risk factors associated with GBC is not understood yet. There is no targeted therapy available for GBC treatment. Hence, understanding the pathogenesis of GBC is urgently needed [6,7]. DNA-based GWAS studies on GBC are limited at present. A few recent studies have reported a potential association between mutation of the SNP variants ABCB1, ABCB4 and DCC with GBC risk at genome-wide level [8]. Furthermore, high throughput whole genome sequence (WGS) analysis has identified a few crucial genes such as TP53, k-ras, EGFR etc., which were reported to be frequently mutated in GBC patients [9,10,11,12].

At present, the most common approach for treating GBC is radical resection. However, the majority of patients with GBC cannot undergo surgical resection due to aberrant clinical manifestations. The symptoms become noticeable in cases where the cancer has already invaded the nearby organs. In such situations, non-surgical therapies such as chemo- and radiotherapy are the only options for treatment. According to the National Comprehensive Cancer Network, single-agent therapy, which includes fluoropyrimidine or gemcitabine-based treatment, and combination therapy regimen, which includes oxaliplatin, cisplatin and capecitabine are the two chemotherapeutic options for GBC patients but both of these are still undergoing clinical trials [13,14,15,16]. The PARP inhibitor olaparib is a novel therapeutic drug that has shown significant improvement in patients with breast cancer and ovarian cancer [17,18,19]. A recent study reported that a GBC patient with a combination of ATM inactivation and STK11 frame-shift mutation showed significant response in inhibiting GBC progression [20]. Till now there is no diagnostic and prognostic biomarker that can detect GBC at the initial stage to potentially select patients who are most likely to benefit from chemotherapy [21].

In recent years, systems approach has been evolving as one of the most promising areas in biology and medicine. Systems-based multidisciplinary approaches can help to understand the complexity of biological systems, as well as contribute to the discovery of novel biomarkers for disease, drug targets and treatments. The advancement of high throughput next generation sequencing (NGS) strategies such as transcriptome sequencing has helped to generate benchmarked cancer-based datasets. Integrative analysis of such datasets using network systems biology approaches provided a basis for investigating biomolecules, and their pathological functions in malignancies. This can further help to determine their potential role in developing efficient cancer treatment strategies [22,23]. Weighted gene co-expression network analysis (WGCNA) is a frequently used systems biology-based method for determining the gene-gene correlation across samples that can be used to identify modules containing clusters of highly correlated gene networks [24].

To this end, we have carried out analysis of GBC RNAseq dataset downloaded from NCBI-GEO database. We have identified the potential genes and TFs associated with GBC progression and pathogenesis through co-expression network analysis of normal and GBC samples followed by transcriptional regulatory network analysis. Functional enrichment analysis and EMT score calculation has also been carried out to identify crucial genes for GBC pathogenesis.

## 2. Materials and Methods

### 2.1. Retrieval of GBC RNA-seq Dataset

A comprehensive and thorough search was conducted in the NCBI database for relevant RNA-seq dataset on GBC. The datasets were checked carefully to be considered for our study based on the following criteria: (i) the dataset must include case-control studies, (ii) the dataset must be paired end and (iii) the sequencing platform for generating the data and experimental protocol should be described in details. Based on the above-mentioned criteria, we selected GSE139682 from NCBI-GEO database (GEO). The dataset comprised of 20 samples in total obtained through resection surgery out of which 10 samples were of GBC tissues and 10 samples were from normal matched tissue.

The GBC dataset was downloaded from NCBI-GEO database in the SRA format. The SRA reads for each sample were converted to fastq reads using fastq-dump. Quality check of the fastq files was done using FastQC. The reads after quality control were aligned using Hisat2 [25] against the reference human genome *Homo sapiens* (GRCh38). The mapped reads were quantified at the gene level to obtain the count matrix for each gene using featureCounts tool [26]. The DESeq2 tool [27] was used for normalization, and log2 transformation of the count matrix. DESeq2 computes the ratio of each gene count to the logarithmic mean of all read counts for that gene across samples. This method identified differentially expressed genes (DEGs) between disease and control conditions. The GBC count data was normalized and transformed in DESeq2 input format, and the level of shrinkage of each gene and the overall covariates were estimated using dispersion plot and principle component analysis respectively. Finally, the significant DEGs of GBC were sorted by considering *p*-adjusted value < 0.05.

### 2.2. Differential Gene Co-Expression Network Analysis

The gene co-expression network gives cluster of genes that are highly correlated. In comparison to other biological network analysis methods, differential co-expression networks can be used to build condition specific sub-networks [28]. The significant DEGs were used as input to build gene co-expression network using the R package WGCNA [24]. Using WGCNA, two weighted gene co-expression networks were constructed for cancer and control conditions. For each cancer and control dataset, Pearson’s correlations analysis of each gene pair was used to build an adjacency matrix using the adjacency function of the WGCNA package. Subsequently, the adjacency matrix was used to create a scale-free co-expression network based on a soft-thresholding parameter βeta (β) to enrich strong correlations between gene pairs [29]. The calculated adjacency matrix was converted into Topological Overlap Matrix (TOM) by using the function TOMsimilarity. This topological overlap matrix was then used as an input for performing hierarchical clustering using the flashClust function for module identification. Finally, the network modules for cancer and control dataset were identified using dynamicTreeCut (an R package) with a minimum module size (minClusterSize) = 30, and minimum sensitivity (deepSplit) = 2 for the gene dendrogram.

### 2.3. Module Preservation Analysis

Preservation analysis was performed to assess the non-preserved module between the cancer network and control network. The basic statistics behind module preservation is to evaluate the preservation of genes within a module by comparing a test network (cancer) with a reference network (control) [24]. It is assumed that the genes embodied in non-preserved modules of cancer network might play a role in the pathogenesis process as compared to the control network. The preservation analysis was carried out using the WGCNA function modulePreservation to determine the connectivity and weight of each module of cancer and control network. The Preservation analysis statistics—*Z-summary* and *medianRank* gives overall significance of the preservation of a module based on degree and connectivity. The *Z-summary preservation* < 2 indicates no preservation, 2 ≤ *Z-summary* ≤ 10 suggests weak to moderate preservation, and *Z-summary preservation* > 10 implies strong preservation [24]

### 2.4. Gene Ontology and Pathway Analysis of the Non-Preserved Module

For interpreting the biological role of significant DEGs identified from non-preserved modules, functional enrichment and pathway analysis was performed using DAVID [30]. The significant DEGs for GBC were used for the GO analysis and KEGG pathway analysis for identification of important cellular processes and pathways in GBC. The top five GO terms for biological processes and KEGG pathway terms were estimated with *p*-value < 0.05.

### 2.5. Screening of Hub Genes from Non-Preserved Modules through Intramodular Connectivity and PPIs Network Analysis

In network concept, connectivity is generally considered as the degree of nodes in the network. In this study, we have used the intramodular connectivity approach for the screening of hub genes within weakly preserved modules. The intramodular connectivity measures the degree of each gene within a module. The criteria used for this study was to calculate the connectivity from the whole network (kTotal) and the connectivity within modules (kWithin). This measure of connectivity is useful to determine the biologically significant modules by calculating the degree of genes within modules.

STRING database (version 10.0) [31] was used to predict potential interactions among candidate genes at the protein level. A combined score of >0.4 was considered to be significant. The protein-protein interaction (PPI) networks of the non-preserved modules were created using the NetworkAnalyst tool [32]. The genes with high number of connections with other genes/proteins were considered as hub genes.

### 2.6. Gene Regulatory Network (GRN) Analysis

From the gene regulatory network (GRN) information regarding the regulatory interactions between transcriptional regulators and their target genes can be obtained [33]. Transcription factors are the key players in regulatory network interactions as they influence gene expression by binding to the start site of the gene promoter region. We have used the significant DEGs as input to construct the regulatory network. The human TFs and their position weight matrices (PWMs) were downloaded from the cis-BP database [34]. The matrix-scan tool were used to predict the interaction between the TFs and its target genes. The results of the matrix-scan were filtered by setting a *p*-value cut off 10^−4^. The TG-TF interactions data along with their prediction scores were represented in the form of interactive network using the Cytoscape software [35]. Considering highest degree centrality, the top six hub transcription factors (TFs) were identified.

### 2.7. EMT Scores Calculation

Epithelial-mesenchymal transitions (EMTs) play a critical role in cancer, particularly in cancer metastasis, apoptotic inhibition, and therapeutic drug resistance, which ultimately effects the overall survival of cancer patients. In this study, we have quantified the EMT scores for each sample using three different scoring metrics—76 Gene Signatures (76Gs), Multinomial Logistic Regression (MLR) and Kolmogorov Smirnov test (KS) [36,37,38]. In 76Gss, the higher the score of EMT, the more epithelial (E) the sample is, whereas, in case of KS and MLR, the higher the EMT scores, the more mesenchymal (M) the sample is.

## 3. Results

### 3.1. Differential Gene Expressions in Gallbladder Cancer

**Figure 1:**
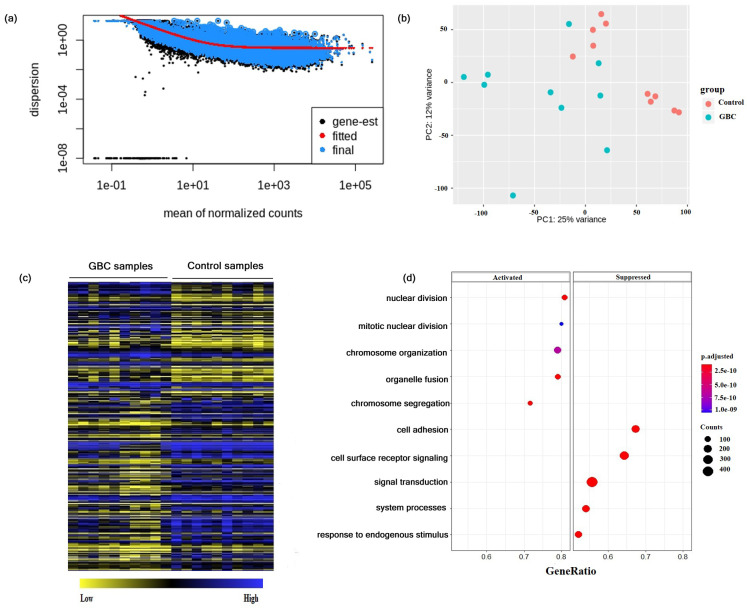
Differential gene expression in GBC. (**a**) An estimate of the dispersion plot for mean of normalized counts. (**b**) Principle component analysis of 10 GBC and 10 adjacent control samples. (**c**) Hierarchical clustering of top 100 significant DEGs in GBC compared to that of control. (**d**) Significant biological processes associated with GBC.

To identify the differentially expressed molecular signatures in GBC, we have carried out analysis of transcriptomic data from 10 tumor samples and 10 adjacent control samples of GBC patients. The resulting data were normalized, and the transformed data were visualized using dispersion and principal component analysis (Figure 1a,b). Two separate clusters for GBC and control samples were identified in the PCA plot (Figure 1b). However, the PCA plot showed that three control samples were diverted towards the GBC cluster. This could be due to invasion of the adjacent control samples by cancer cells in GBC patients. From the differential gene expression analysis, 2980 significant DEGs were identified in GBC as compared to that of controls by taking Padj ≤ 0.05. Hierarchical clustering analysis of the significant DEGs showed that the GBC and the adjacent control samples exhibited differential gene expressions (Figure 1c). The significant DEGs identified in GBC are largely linked to the cell cycle regulation and signal transduction processes (Figure 1d). This suggests that genes related to cell cycle progression and checkpoint regulatory proteins might be crucial for GBC development. 

### 3.2. Construction of Gene Co-Expression Network and Module Detection

**Figure 2:**
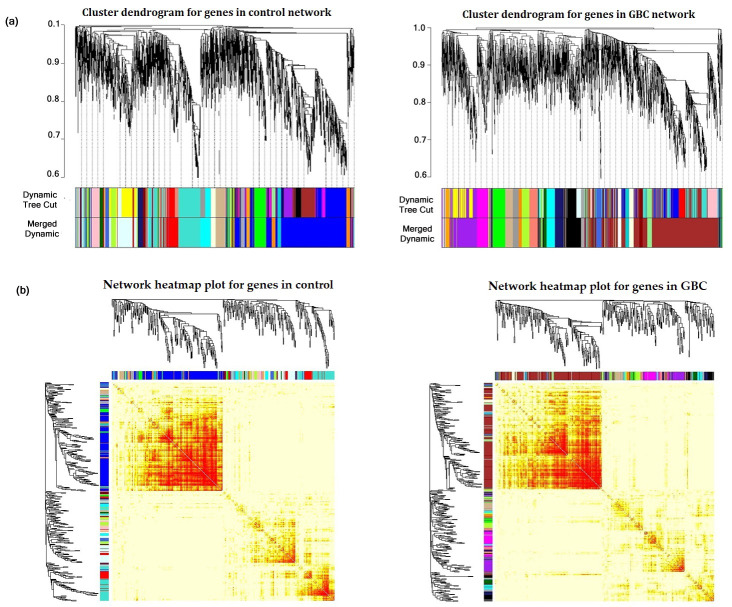
Construction of differential gene co-expression networks. (**a**) Clustering dendrogram of modules in control network and GBC network respectively. (**b**) Clustering dendrogram heatmap plot with module colors based on topological overlap for control and GBC network respectively.

For performing differential gene co-expression network analysis, the log2 transformed gene expression values of significant 2980 DEGs for 10 GBC samples and 10 adjacent control samples identified through DESeq2 were considered. The differential co-expression networks for GBC and control conditions were constructed separately using WGCNA package in R. The co-expression network construction needs the selection of a soft thresholding power β for satisfying scale-free topology of the network. The soft-thresholding power β for GBC and control were 18 and 20, respectively (Supplementary Figure S1). Subsequently, the modules with clusters of highly connected genes were identified using hierarchical clustering approach. The branches in cluster dendrogram correspond to modules containing genes of high connectivity. A total of 18 and 20 modules were identified in control and GBC condition respectively. The cluster dendrogram containing modules and heatmap plot for control and GBC network is represented in Figure 2.

### 3.3. Detection of Non-Preserved Module from GBC and Control Co-Expression Network

**Figure 3:**
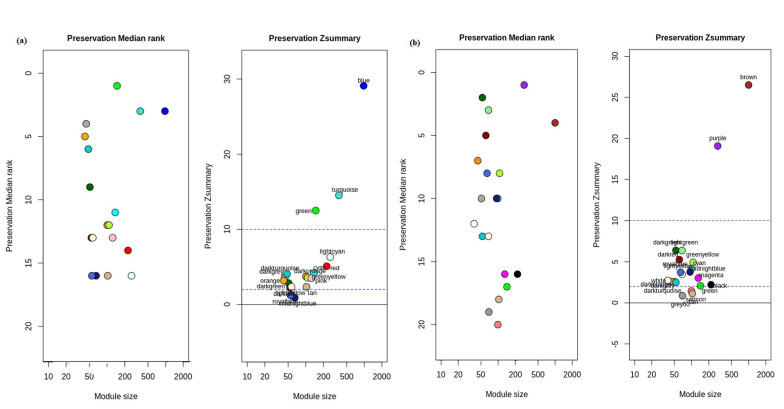
Preservation analysis of modules based on Z-summary and medianRank. (**a**) Identification of modules in the control condition. The modules in midnightblue and royalblue color are non-preserved. (**b**) Identification of modules in the GBC condition. The modules in tan, salmon and grey60 color are non-preserved.

The module preservation analysis from differential co-expression network was performed to identify non-preserved modules in control and GBC condition using statistical measures- *Z-summary* and *medianRank*, which calculate the extent of preservation based on the connectivity of genes in each module. In this study, module preservation analysis was performed by the following approaches: (i) GBC vs. control, where the cancer data was considered as the test data and the reference data was the control data; (ii) control vs. GBC, in which the control data was considered as the test data and the GBC data was the reference data. The identification of non-preserved modules in both control and GBC networks has been represented in Figure 3. The non-preserved modules give insights of distinct molecular signatures in GBC modules compared to that of control modules.

In GBC to control module preservation analysis, three modules, salmon, tan and grey60 were identified as the non-preserved module in GBC with *Z-summary*—1.4, 1.1, 0.86 and *medianRank*—20, 18, 19 respectively (Supplementary Table S1). For control to GBC preservation analysis, two non-preserved modules were detected which were midnightblue and royalblue with *Z-summary* preservation—0.91, 1.2 respectively. The *medianRank* of both midnight and royalblue modules was 16 (Supplementary Table S2).

### 3.4. Hub Gene Identification from Non-Preserved Modules

The genes having high degree of connectivity or high correlations in significant modules were regarded as hub genes. For hub gene identification, we have considered the non-preserved modules identified from both GBC and control networks, and determined their topological measure with respect to intra-modular connectivity. In total five genes were considered as potential candidates in terms of correlation weight (degree). The weight of the potential candidate genes identified through intra-modular connectivity analysis is given in Table 1. The genes with highest intra-modular connectivity from each module (hub gene) were AL009178.3 (a novel transcript), ADAM metallopeptidase domain 18 (ADAM18), mitogen-activated protein kinase 15 (MAPK), lethal 3 malignant brain tumor-like protein 1 (L3MBTL1) and alkaline phospatase, placental-like 2 (ALPPL2).

**Figure 4:**
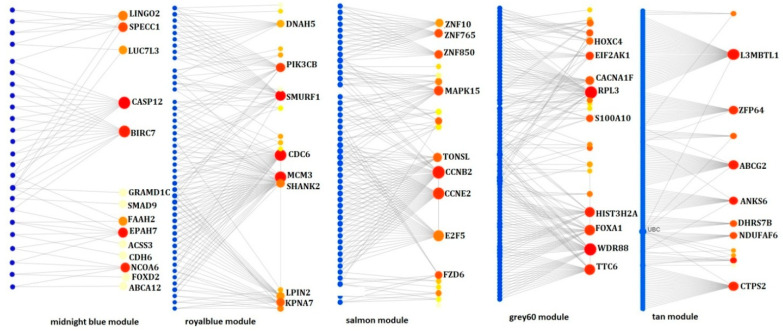
PPI network analysis of non-preserved modules. The PPIs network of the genes identified from the non-preserved modules of control network (mignightblue and royal blue modules) and GBC network (salmon, tan and grey60 modules). The small blue circles represent the proteins and large red node represents the genes in the modules.

Subsequently, PPI networks for the genes in each of the non-preserved modules were constructed, as shown in Figure 4. These genes were—baculoviral IAP repeat-containing protein 7 (BIRC7), cyclin B2 (CCNB2), cell division cycle 6 (CDC6), L3MBTL1 and WD repeat domain 88 (WDR88). All the identified were found to be upregulated in GBC, compared to the controls. This indicates that upregulation of these hub genes might drive GBC development and progression. The top hub genes with degree centrality identified through PPI network analysis is given in Table 2.

### 3.5. Functional and Annotation and Pathway Associated with Genes of the Non-Preserved Modules

The functional GO terms and pathways associated with the gene modules were identified using DAVID tool. The statistical significance of *p*-value < 0.05 were considered for determining the important biological processes and KEGG pathways related to GBC progression. The functional annotation analysis identified that the module genes were mostly associated with cell cycle regulation processes, metabolic pathways and signal transduction processes. The top ranked significant biological processes and pathways are tabulated in Table 3; Table 4, respectively.

### 3.6. Identification of Hub Transcription Factors in GBC through TG-TF Regulatory Network Analysis

Out of the 2980 DEGs, 106 DEGs code for transcription factors (TFs). Considering these TFs as the source nodes and the DEGs (including the TFs) as the target nodes, we created a transcriptional regulatory network. The degree distribution did not follow a Poisson distribution (mean of degree distribution = 78.88603; variance of degree distribution = 41,142.97) and hence, the network was not a random network. The topological parameters of the network such as clustering coefficient, path length, network assortativity were calculated using the R package igraph. The assortivity of the network was negative i.e., −0.1024318, meaning the nodes with higher degrees tend to interact with nodes of smaller degrees. This is in compliance with the observation that real-world networks tend to have negative assortivity [39]. The degree coefficient Ɣ, of the degree distribution was calculated to be 5.467 and a power-law was fitted in the distribution. The hub TFs identified in GBC were PAX6, KLF15, NR2F1, TFAP2C, FOXJ2 and FLR. Among these, PAX6, TFAP2C and FOXJ2 were present in modules identified from GBC co-expression network.

### 3.7. EMT Analysis Identified Differential EMT Patterns in Hub Transcription Factors

**Figure 5:**
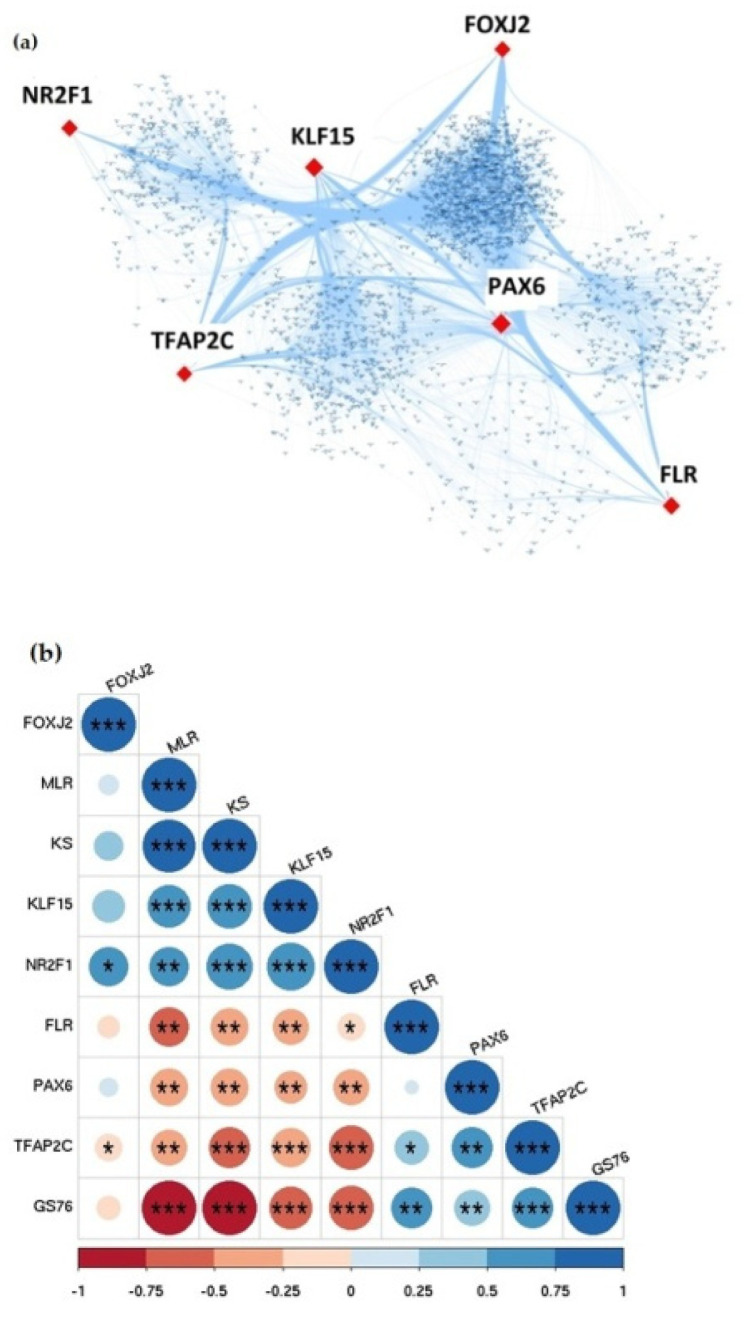
Transcriptional regulatory network analysis of DEGs in GBC. (**a**) Identification of hub TFs in GBC based on degree centrality. The red node represents the top hub TFs and small blue nodes represent target genes. (**b**) Pairwise correlation of EMT score of hub TFs identified through TF-TG interactions. The significance of each hub TFs is represented with a symbol- *p*-value < 0.001 (***); *p*-value < 0.01 (**) and *p*-value < 0.05 (*).

Next, we quantified the extent of epithelial-mesenchymal transition (EMT) that the GBC samples had gone through. We used three different algorithms (76GS, KS, MLR) that score the degree of EMT in transcriptomic data—while higher KS and MLR scores indicate a more mesenchymal state, whereas a high 76GS score indicates a more epithelial phenotype (Supplementary Table S3). Based on previous observations, 76GS scores are expected to correlate negatively with KS and MLR scores for GBC samples. [40]. Indeed, we observed a positive significant correlation between MLR and KS scores with both the scores were negatively correlated with 76GS scores. This consistency indicates that the EMT scoring methods well recapitulate the extent of EMT in GBC samples. Further, we examined how the six hub TFs identified in GBC were associated with coordinating a more epithelial vs. a more mesenchymal phenotype. Levels of KLF15 and NR2F1 associated with a more mesenchymal state (i.e., positive correlation with KS and MLR scores and negative correlation with 76GS scores). FOXJ2 also showed similar trends but they were not statistically significant. On the other hand, FLR, PAX6 and TFAP2C were associated with an epithelial state (i.e., positive correlation with 76GS scores, and negative correlation with KS and MLR scores). Thus, the six hub TFs identified in GBC were associated differentially with epithelial vs. mesenchymal status. They seem to form two ‘teams’ of players—one promoting EMT, the other set inhibiting EMT. The hub TFs identified from TF-TG regulatory network and its association with EMT event in GBC has been illustrated in Figure 5.

## 4. Discussions

Gallbladder cancer is a fatal malignancy of the biliary tract system. Standard molecular screening of GBC is of the utmost necessity for detecting the onset of GBC at an early stage and to reduce the mortality rate of patients suffering from GBC. Next-generation sequencing techniques are being widely used in cancer-related studies. However, omics-based studies have been limited in GBC due to the rarity of the disease. The therapeutic strategies of GBC are also limited due to the lack of specific molecular targets. Hence, the main objective of this study was to identify important genes and TFs potentially related to GBC pathogenesis.

To this end, we have used an integrative systems-based approach to identify hub genes (potential biomarkers) in GBC. Here we have analyzed a transcriptomic dataset consisting of 10 GBC and 10 adjacent control samples. In total, 2980 significant DEGs were identified in GBC samples compared to the controls. The identified DEGs were then used to construct differential gene co-expression networks and to determine significant co-expressed modules in both GBC and control samples. The functional annotations and KEGG pathway analysis were further evaluated to identify significant biological processes and pathways enriched in non-preserved modules. We analyzed and identified the hub transcription factors from significant DEGs that might have important role in gene regulation process during GBC development. The hub genes identified from the non-preserved co-expressed modules were largely associated with cell cycle machinery and signaling processes.

The cell cycle machinery is a highly regulated and intricate process which governs cell growth, cell proliferation and cell division through its regulatory genes. Cell cycle regulatory molecule mostly involves growth-regulatory signaling proteins- CDK and CDKI and associated genes/proteins that check for any anomalies throughout the genome. Disruption in the regulation of cell cycle machinery/components are frequently observed in several malignancies where it contributed to malignant transformation and resistance to cancer drugs [41,42]. Numerous studies have reported the significance of cell cycle aberration towards human cancer development. The cell cycle defects in cancer mainly involve uncontrolled proliferation through dysregulation in any of its cell cycle components either due to CDK function mis-regulation, and/or decrease in the negative regulator of CDKI [43,44]. The most important component of the cell cycle machinery is the DNA replication initiation process and pathway. The DNA synthesis process acts as a relay system of the cell cycle process that connects various growth signaling network with DNA replication complex and therefore this component serves as an important diagnostic and prognostic target [45]. The DNA replication and the mitotic process regulation are considered to be the central players involved in these cell cycle phase transitions and therefore they not only are useful cancer biomarkers, but also acts as potent targets for mechanism-based therapies [46], but the initiation of oncogenesis process is not only associated with cell cycle components alone. The development of malignant tumors involves mis-regulation of the cell death machinery and cell–cell and/or cell–matrix interactions that co-operate with cell cycle defects [47].

The hub genes identified through differential gene co-expression networks analysis followed by PPIs analysis were directly or indirectly associated with components of the cell cycle system, apoptotic regulation and cell-cell adhesion process that ultimately give rise to uncontrolled cell proliferation and later on to a full bloom malignancy. The hub genes L3MBTL1, MAPK15, CCNB2 and CDC6 are crucial elements that act as a control system for coordinated regulation of cell cycle system. L3MBTL1 is known to be a potential tumor suppressor gene in *Drosophila* fly. It binds to the chromatin complex during S-phase of the cell cycle and also regulates the target genes of E2F-RB negatively that are necessary for S-phase initiation. L3MBTL1 was reported to be associated with breast cancer and myeloid leukemia including AML [48,49,50]. The family of MAPK proteins plays a key role in different cellular events such as cell differentiation, cell growth and development, cellular transformation and apoptosis. It involves a sequence of protein kinase signaling cascade which is important for regulation of cellular proliferation [51]. MAPK15 is known to be an important extracellular signal transducing kinase which is known to be activated by human serum. The MAPK15 gene is unique as it does not have specific MEK upstream regulation like other MAP kinases. The activity of MAPK15 is found to be modulated by several oncogenes. Recent study reported the association of MAPK15 with BCR-ABL mediated autophagy and its role in oncogene dependent cancer cell proliferation and progression [52,53]. CCBN2 has been found to be linked with poor survival outcome in gastric and hepatocellular cancer [54,55]. CDC6 also acts as a crucial player in cell cycle system and acts as a replication licensing factor and governs the DNA replication process through maintenance of the cell cycle checkpoints machinery. CDC6 is found to be reported in initial stages of many cancers and also contributes to the oncogenic activities in tumor development [56,57]. Aberrant CDC6 expression is reported to be associated with several malignancies [58].

ADAM18, a membrane anchored gene (matrix metalloproteinase) of the ADAM family proteins regulates cell adhesion via interaction with integrins. It plays an important role in the release of biologically important ligands, such as tumor necrosis factor-alpha, epidermal growth factors, transforming growth factor-alpha, and amphiregulin [59]. In human cancer, overexpression of specific ADAMs is related to tumor progression and poor outcome. Therefore, it is regarded as a potential target for cancer therapeutics, particularly those cancers that are human epidermal growth factor (EGFR) receptor (EGFR) ligands or TNF-alpha positive [60,61].

ALPPL2 belongs to the member of the ALPP alkaline phosphatases which are reported to be associated with tumor initiation. It was reported as a specific and targeted tumor cell surface antigen. It is significantly associated with gastric cancer and pancreatic cancer and also acts as a novel protein in pancreatic cancer [62,63].

BIRC7, a novel member of the IAP family, is found to be highly overexpressed in various cancer types. BIRC7 was found to be overexpressed in 66% of the cancers and to be absent in normal cells/tissues. The function of BIRC7 gene is mainly related to apoptotic regulation and signaling processes. The overexpression of BIRC7 in cancers is reported to be associated with cancer drug and radiotherapy resistance, disease recurrence and poor survival [64,65]. Moreover, increased expression of BIRC7 was found in extrahepatic cholangiocarcinoma and was significantly associated with poor prognosis and overall survival of the patient [66]. WD repeat domain 88 (WDR88) present on chromosome 19 is known to be important biomarker for early prostate cancer development. This gene is evolutionarily conserved and can found in 167 organisms as an orthologous gene. Hence this gene might act as an important target in GBC [67].

We observed that genes related to cell cycle regulatory and signal transduction processes were essential and significant in pathogenesis of GBC. The genes and TFs identified from the non-preserved modules may play key roles in the pathogenesis of GBC. The identified hub genes provided the basis for further in-depth studies for development of prognostic, diagnostic and therapeutic biomarkers. In summary, this study used differentially co-expression network analysis and transcriptional regulatory network analysis to identify key hub genes associated with GBC pathogenesis.

## Figures and Tables

**Table 1:** Identification of hub genes from non-preserved modules in GBC and control condition through Intramodular connectivity approach.

Control to GBC				GBC to Control					
Midnightblue		Royalblue		Salmon		Tan		Grey60	
Gene	Weight	Gene	Weight	Gene	Weight	Gene	Weight	Gene	Weight
AL009178.3	2.87	ADAM18	2.87	MAPK15	12.51	L3MBTL1	11.38	ALPPL2	8.87
SPATC1	2.71	CNTN4	2.71	TRAPPC9	12.08	ZNF337-AS1	10.47	PATE4	8.01
CTSV	2.70	NUP62CL	2.70	OPLAH	11.72	AC099661.1	9.64	AP00842.3	7.85
AL353746.1	2.64	QTRT2	2.64	OTUD6B	10.97	AC240565	8.68	GPATCH1	7.80
AL360270.1	2.61	LINCO1517	2.61	TAF2	10.72	C1QTNF	8.14	AP000977.1	7.06

**Table 2:** Hub gene identification from non-preserved modules through PPI network analysis.

Control to GBC				GBC to Control					
Midnightblue		Royalblue		Salmon		Tan		Grey60	
Gene	Degree	Gene	Degree	Gene	Degree	Gene	Degree	Gene	Degree
CDC6	30	BIRC7	12	CCNB2	30	L3MBTL1	34	WDR88	31
MCM3	28	CASP12	11	CCNE2	25	ABCG2	23	RPL3	31
SMURF1	22	UBC	6	E2F5	22	CTPS2	12	TIC6	23
PIK3AB	17	EPH7	6	MAPK15	15	SIM2	7	FOXA1	22
SHANK2	14	LINGO2	5	TONSL	12	PTP4A1	7	HIST3H2A	18

**Table 3:** Enriched biological processes associated with non-preserved modules identified from GBC network.

Modules	Biological Processes (GO Terms)	Counts	Genes	*p*-Value
midnight blue	intracellular signal transduction	4	NEK11, DGKB, GUCY1B2, NUDT4	0.0390
royalblue	negative regulation of DNA replication	2	S100A11, CDC6	0.0390
salmon	dorsal/ventral axis specification	2	PAX6, RGS20	0.0370
	neuron migration	3	CELSR3, PAX6, PTK2	0.0420
	negative regulation of keratinocytes proliferation	2	CTSV, EPPK1	0.0430
	interphase of mitotic cell cycle	4	CCNB2, CCNE2, E2F5, TAF2	0.0382
grey60	Negative regulation of translation	2	FXF1, EIFAK1	0.00824
	Cell fate commitment	3	FOXA1, HOXA11, TFAP2C	0.0131
	Developmental growth		HOXA11, TFAP2C, PLAC1	0.0186
tan	planar cell polarity pathway involved in axon guidance	2	VANGL2, RYK	0.0120
	Epidermal cell differentiation	2	OVOL2, SPINK5	0.0160
	Negative regulation of serine type endopeptidase activity	2	SPINK1, SPINK5	0.0280

**Table 4:** Enriched KEGG pathways associated with non-preserved modules identified from GBC network.

Module	KEGG Pathways	Counts	Genes	*p*-Value
midnightblue	Glycerolipid metabolism	2	HLA-DMA	0.00683
	Toxoplasmosis	2	DGKB, LIPC	0.0222
	Apoptosis	2	CTSV, CASP12	0.0313
	Glycosaminoglycan degradation	1	HYAL4	0.0382
royalblue	Phosphotidyl inositol signaling	2	PIK3CB, ITPKA	0.00762
	Cell cycle	2	MCM3, CDC6	0.0204
salmon	Cell cycle	3	E2F5, CCNB2, CCNE2	0.0450
	Small cell lungs cancer	2	CCNE2, PTK2	0.0385
	P53 signaling	2	CCNE2, CCEB2	0.0240
	Ubiquine biosynthesis	1	COQ2T	0.0364
grey60	Thiamine metaboilism	1	ALPPL2	0.0266
	Necroptosis	2	H2AW, RNF103-CHMP3	0.0292
	Alcoholism	2	H2AW, H2BO1	0.0355
	Histidine metabolism	1	ALDH3B2	0.0380
tan	Wnt signaling pathway	2	VANGL2, RYK	0.0321
	Steroid biosynthesis	1	CYP24A1	0.0339

## Data Availability

The dataset used for this study is available at NCBI-GEO database (GEO Accession No. GSE139682).

## Supplementary Materials

The following are available online at https://www.mdpi.com/article/10.3390/jcm10163520/s1, Figure S1: β power value for GBC and control network respectively generated through gene co-expression network construction. Table S1: Z-*summary* preservation of modules in GBC network, Table S2: Z-*summary* preservation of modules in control network, Table S3: Pairwise correlation analysis of each sample using three EMT scoring metrices- KS, MLR and 76GS (negative strong correlation between 76GS and KS or MLR, positive strong correlation between MLR and KS).
