# Supplementary material for: An Integrative Systems Biology Approach Identifies Molecular Signatures Associated with Gallbladder Cancer Pathogenesis
Source: J Clin Med. 2021 Aug 10;10(16):3520. doi: 10.3390/jcm10163520 (PMC8397040; doi:10.3390/jcm10163520)
Supplement: Supplementary file 1 [file jcm-10-03520-s001.zip › jcm-1228419-supplementary.pdf]

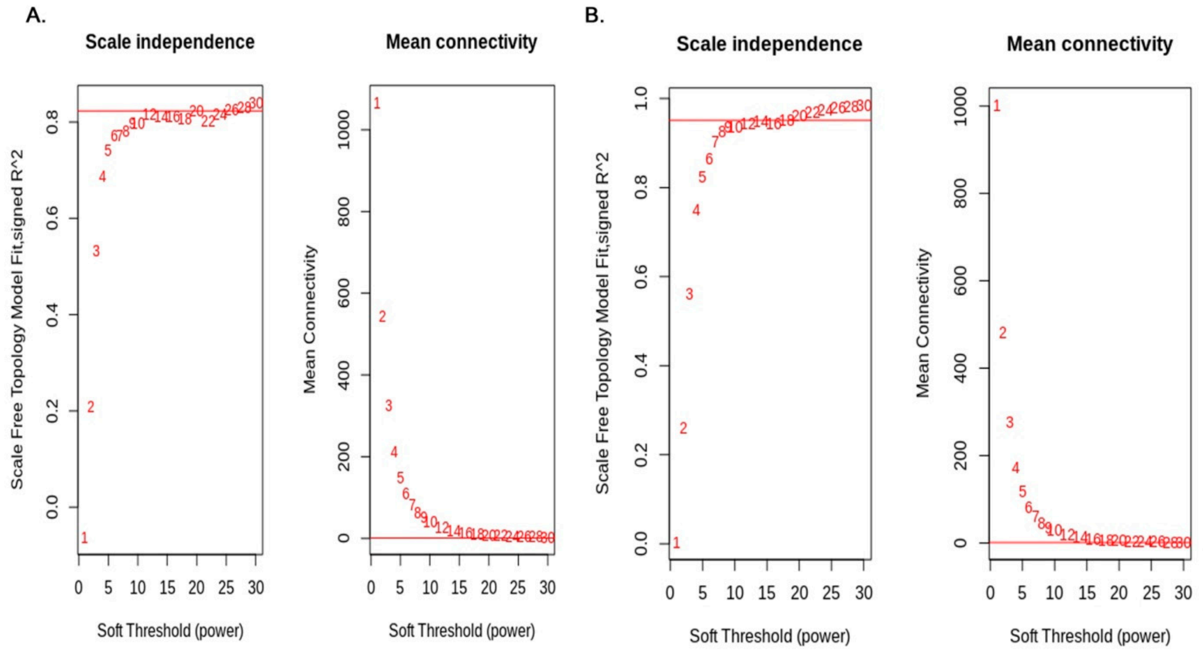

Figure S1. β power value for GBC and control network generated through gene co-expression network construction respectively.

Table S1. Z-*summary* preservation of modules in GBC network.

Module	*medianRank*.pres	*Z-summary*.pres
black	16	2.2
brown	4	27
cyan	10	4.1
darkgreen	2	6.4
darkgrey	10	2.6
darkorange	7	2.6
darkred	5	5.3
darkturquoise	13	2.5
green	17	2.1
greenyellow	8	4.9
grey60	19	0.86
lightgreen	3	6.4
lightyellow	13	3.5
magenta	16	3
midnightblue	10	3.8
purple	1	19
royalblue	8	3.7
salmon	20	1.4
tan	18	1.1
white	12	2.7

Table S2. Z-*summary* preservation of modules in control network.

Modules	*medianRank*.pres	*Z-summary*.pres
blue	3	29
cyan	11	4.2
darkgreen	9	2.8

darkgrey	4	3.6
darkorange	12	3.7
darkred	13	2.2
darkturquoise	6	4.1
green	1	12
greenyellow	12	3.6
lightcyan	16	6.3
lightyellow	13	2.3
midnightblue	16	0.91
orange	5	3.2
pink	13	3.5
red	14	5.1
royalblue	16	1.2
tan	16	2.3
turquoise	3	15

Table S3. Pairwise correlation analysis of each sample using three EMT scoring metrices- KS, MLR and 76GS (negative strong correlation between 76GS and KS or MLR, positive strong correlation between MLR and KS).

Sample_name	76Gs	MLR	KS
GSM4146148	6.92582495614312	0.922485283	−0.419032672
GSM4146149	−15.4277105379992	1.038697965	0.272364103
GSM4146150	−2.78710068072522	0.956055384	-0.085746897
GSM4146151	3.08850458758825	0.939545828	−0.253673848
GSM4146152	3.54856723716013	0.940623403	−0.276644314
GSM4146153	−11.2641449810022	0.962427526	0.121129976
GSM4146154	7.92566592889314	0.931538294	−0.382222856
GSM4146155	4.85039097543316	0.924028816	−0.223997717
GSM4146156	5.34065727847344	0.920960796	−0.30960194
GSM4146157	5.83634613093576	0.935251049	−0.229847339
GSM4146158	11.6216471377933	0.887363848	−0.330860322
GSM4146159	−19.0938301382121	1.06273886	0.357968326
GSM4146160	12.4611940094735	0.932319314	−0.486517335
GSM4146161	6.64607743670175	0.942107475	−0.256955343
GSM4146162	11.4271108425853	0.903626614	−0.482950492
GSM4146163	−18.4380535030102	1.046387984	0.277928378
GSM4146164	11.1470869283106	0.903418062	−0.58952775
GSM4146165	3.95593866468601	0.925905771	−0.212726495
GSM4146166	−9.47737699957706	1.038349726	0.130831788
GSM4146167	−18.2867952736515	1.038623957	0.247253531
